# Correlations of Glycaemic Index and Estimated Whole Blood Viscosity with Blood Cell Indices in Diabetes Mellitus Management: A Clinical Laboratory Medicine Observational Cohort Study †

**DOI:** 10.3390/jcm15020892

**Published:** 2026-01-22

**Authors:** Jovita I. Mbah, Phillip T. Bwititi, Prajwal Gyawali, Lin K. Ong, Ezekiel U. Nwose

**Affiliations:** 1 School of Health & Medical Sciences & Centre for Health Research, University of Southern Queensland, Toowoomba, QLD 4350, Australia; 2 School of Dentistry & Medical Sciences, Charles Sturt University, Wagga Wagga, NSW 2678, Australia

**Keywords:** HbA1c, eWBV, clinical laboratory medicine, RBC indices, platelet ratios, lymphocyte ratios

## Abstract

**Background/Objective**: The risk of bleeding is part of blood flow pathophysiology in diabetes mellitus (DM), and there may be potential for the relationship between blood cell indices and estimated whole blood viscosity (eWBV) in DM. However, red blood cell (RBC) indices, platelet ratios, and lymphocyte ratios have been part of routine haematology tests in clinical medicine including diabetes management. This study investigated two research questions. Firstly, how does eWBV correlate with RBC indices, platelet ratios, and lymphocyte ratios? Secondly, which parameters of RBC in routine full blood count (FBC) correlate more with glycated haemoglobin (HbA1c) changes? **Methods**: This was a laboratory-based clinical observational cohort study using secondary data from ongoing research. Data collected included FBC and biochemistry (HbA1c and serum protein level). Dependent variables were platelet and lymphocyte ratios as well as eWBV. **Results**: Averages for all parameters in the cross-sectional data were within normal range, except high HbA1c (7.67%) and marginally high monocyte-to-lymphocyte ratio. In the periodic cohort analysis, only RBC distribution width showed a significant difference (*p* < 0.04) between cohort groups, but least correlated with HbA1c changes. Further analysis for correlations among change scores showed that RBC had the strongest positive linearity for HbA1c (r = 0.30) and among the top three for eWBV (r = 0.54), while mean cell volume (MCV) has the strongest inverse for HbA1c (r = −0.47). **Conclusions**: The ongoing clinical use of RBC variables is superior to profiles of platelet and/or lymphocyte ratios in assessing the potential risk of bleeding (i.e., hypo-viscosity) in diabetes.

## 1. Introduction

Whole blood viscosity (WBV) is the inherent resistance to blood flow and is influenced by factors such as red blood cell (RBC) count (haematocrit), RBC’s deformability, and blood proteins levels [1]. WBV is a vital parameter of physiological implications as elevated levels increase the risk of clotting and other cardiovascular diseases (CVD), while a lower level indicates risk of bleeding [2,3]. Yet, the utilisation of WBV in clinical laboratory medicine has not been fully utilised [4,5,6].

In the effort to motivate utilisation of WBV, a formula for estimated WBV (eWBV) was developed by a team in the United States [7]. This eWBV formula has been advanced by the development of an algorithm tool with online and offline models [8]. The eWBV tool has been revalued in a series of studies [9], which includes proof of concept and revalidation studies [2,10]. Indeed, eWBV method has been used in clinical medicine for over 30 years [11].

WBV is elevated in diabetes mellitus through several pathophysiologic mechanisms. In diabetes mellitus, a metabolic disorder characterised by persistent hyperglycaemia, high blood viscosity arises due in part to elevation in glycated haemoglobin (HbA1c). Changes in blood viscosity in diabetes is related to structural changes in haemoglobin molecules, osmotic disturbances, and cytoplasmic viscosity within the cells [12]. This leads to a reduced plasma volume and increased concentration of RBC, thus raising haematocrit (HCT) [13]. Chronic hyperglycaemia and the related oxidative stress cause structural and functional changes in RBC membranes leading to reduced deformability and increased resistance to flow [14]. The combined effects of high blood glucose, plasma proteins, and lipid levels result in increased viscosity [13,15]. These changes are reflected in blood cell counts and indices in routine clinical medicine monitoring for several diseases [12].

Routine full blood count (FBC) is one of the most accessible and simplest laboratory tests for clinical medicine. FBC consists of a panel of tests used to differentiate various diseases such as anaemia, blood flow issues, and inflammation, amongst others. For instance, RBC count, mean corpuscular haemoglobin concentration (MCHC), MCV, mean corpuscular haemoglobin (MCH), and RBC distribution width (RDW) are reviewed for the diagnosis of anaemia [16]. Further, increased RBC count leads to increased WBV and reduced microcirculatory flow leading to CVD complications such as in diabetes [17].

Changes in RBC parameters influence WBV in diabetes and appear to be dependent on the glycaemic status. A study to assess changes in RBC parameters among good versus poor glycaemic controls observed differences in all the RBC parameters. RBC and Hb were higher in good glycaemic control, while MCV, MCH, and RDW were higher in poor glycaemic controls. RBC negatively correlated with HbA1c while MCV, MCH, and RDW were positively correlated with HbA1c [18,19,20]. Another study reported MCHC to have a positive relationship with worsening glycaemia [21]. The rheological changes in diabetes are reflected in various haematological parameters and understanding these changes helps in clinical medicine management including micro and macro complications [22]. Indeed, variations in blood viscosity among diabetic patients have been reported to correlate with the severity of retinopathy and the correlation is attributed to decreased RBC deformability [23].

Platelets are key blood components in repair following vascular injury and activation contributes significantly to the development and progression of atherosclerotic lesions [24]. Platelets and WBC play a vital role in the pathogenesis of ischemic stroke and studies showed that low platelet and high white blood cell numbers, i.e., a low PWR result, in severe ischaemic stroke attack and worsen prognosis [25]. The higher the PWR, the better the clinical outcome [26], and this lowers the risk of diabetes and holds potential in early detection of diabetes [27]. For instance, patients with aneurysmal subarachnoid haemorrhage with PWR < 15.69 are likely to develop post-operative complications [28,29], and subarachnoid haemorrhage has been the focus of PWR research. It is possible that the blood flow pathophysiology involving haemorrhage may be observable in diabetes cardiovascular pathology, hence requires investigation [30]. Platelets in diabetes show increased reactivity and number [31]. In the context of blood transfusion, increased platelet to red blood cell ratio (PRR) has been shown to improve patients’ survival without exacerbating organ failure [32].

RDW is elevated in acute myocardial infarction [33], while elevated RDW to platelet ratio (RPR) reflects the severity of inflammation and is associated with poor prognosis in spontaneous deep-seated intracranial haemorrhage, which is associated with thrombosis [34]. RPR is also postulated to be a predictor of mortality in spontaneous intracerebral haemorrhage [35].

WBC derived ratios such as neutrophil to lymphocyte ratio (NLR) are associated with increased risk of developing diabetic complications. Elevated NLR levels have been reported in diabetes patients with peripheral neuropathy compared to diabetics without neuropathy [36]. Moreover, an increase in apparently healthy individuals may indicate underlying impairment of glucose metabolism [37]. NLR has been presented as a potential indicator of vascular complications and poorer outcome in diabetic patients [38].

Other differential white cells, viz monocytes, are associated with increases in blood viscosity in diabetes indirectly through their role in chronic inflammation and development of related complications [39], The monocyte-to-lymphocyte (MLR) ratio is an inflammation marker elevated in diabetes signalling increased risk and severity for complications like nephropathy, neuropathy, and CV events [40]. In diabetes, elevation in MLR and viscosity are associated with underlying inflammation and vascular complications of the disease. While MLR is an inflammatory marker and viscosity is a hemorheological factor, both serve as indicators of increased CV risk and poor outcome in diabetic patients [13,40].

Platelet-to-lymphocyte ratio (PLR) is another white cell index reported as a useful indicator in diagnosis of disease. Positive correlation of PLR was found in diabetic complications and was described as diagnostic for diabetic nephropathy and diabetic retinopathy [41,42]. Therefore, there exists concepts of platelet ratios (PRR, RPR, and WPR) and lymphocyte ratios (MLR, NLR, and PLR), which have yet to be commonly assessed in clinical laboratory medicine compared to the RBC indices (MCV, MCH, and MCHC).

Statement of problem: Although HbA1c has been established as the gold standard for monitoring glycaemia in diabetes, readily available blood indices may serve as a cost-effective complement in identifying individuals at risk of diabetic blood flow complications. The various factors (physiological and pathophysiological) that influence WBV are known and described [43]. It suffices that although changes in RBC parameters influence WBV and are dependent on HbA1c [18,19,20], the correlations and/or differences between these changes with that of WBV and HbA1c are unknown.

It is therefore pertinent to investigate the relationship between WBV and blood cell indices in glycaemia with a view to mitigating the development of diabetic complications. It is pertinent to note that over the past two decades, work has been ongoing regarding WBV in diabetes and a review of these was recently published [9]. The focus of this work is adding and advancing to what has already been achieved. That is, given the emerging research interests on platelet ratios, and lymphocyte ratios, it is relevant to consider the comparison with RBC indices, whether the ratios matter clinically, and if it is necessary to translate into clinical medicine for diabetic patients during their intervention?

Objective: To investigate the relationship between blood cell indices and estimated whole blood viscosity (eWBV) among individuals attending diabetes mellitus monitoring.

Research questions:

1. How does eWBV correlate with the indices of RBC, platelet, and WBC?
2. Which of the blood cell counts and indices rank most strongly correlated with changes in HbA1c?

## 2. Materials and Methods

***Design***: This was a clinical laboratory-based observational study involving retrospective longitudinal analysis on secondary dataset based on published work. The study design could also be viewed as cohort and correlational based on analysis.

***Setting***: Orange area in regional New South Wales (NSW) of Australia. The private general practice (Wellness Centre) in Orange, regional NSW. Laboratory data of patients who visited this centre were collected.

***Participants***: De-identified pathology data of diabetes patients from the General Practice represented the participants. This involved no contact with individual participants.

***Selection criteria***: Inclusion criteria were data that had at least a pair of results to allow for cohort analysis and exclusion criteria were incompleteness of data.

***Data variables***: 18 laboratory variables were collected from the laboratory information system. The variable comprised eight (8) independents and ten (10) dependents.

❖ The independent variables included haematocrit, haemoglobin, and RDW; as well as cell counts of platelet, RBC, and WBC—i.e., six independent variables from FBC. The other two independent variables were serum total protein levels and HbA1c.
❖ The dependent variables: eWBV was derived from haematocrit and serum total protein levels, based on the published formula [7,8]. The other nine dependent variables with associated references included:
  - RBC indices as reported on routine FBC:
    - MCV;
    - MCH;
    - MCHC.
  - Platelet count indices:
    - Platelet/RBC ratio (PRR) [30,44];
    - Platelet/WBC ratio (PWR) [25,26,28,29];
    - RDW/platelet ratio (RPR): thrombosis and haemorrhage [35].
  - WBC ratios, more specifically, lymphocyte ratios [45]:
    - Neutrophil/Lymphocyte ratio (NLR);
    - Platelet/Lymphocyte Ratio (PLR);
    - Monocyte/Lymphocyte Ratio (MLR).

***Study size***: Sample data, N = 244 that had at least two data points of results to allow for periodic cohort analysis fulfilled the inclusion criteria.

***Grouping***: Two grouping analyses were performed based on the dichotomous categorisation of paired HbA1c data points of results into high and low periodic groups.

***Statistical analysis***: This study was designed to follow a mixed-methods approach. It included a cross-sectional evaluation and periodic cohort analysis as explained below.

Cross-sectional: This was a descriptive evaluation of eWBV and indices of RBC (MCV, MCH, MCHC), platelets (PRR, PWR, RPR), and WBC (re: lymphocyte ratios—MLR, NLR, PLR). Afterwards, the correlations among variables were evaluated.

Periodic cohort: Paired cohort analysis included univariate *t*-tests and change score analysis. Rather than evaluating causality, this was aimed at assessing the strength of change within the individual [46]. The paired cohort analysis controlled for gender because every pair represented the same participant. The hypothesis tested in this cohort evaluation was ‘no statistically significant difference in any of the variables’.

In both the cross-sectional and periodic cohort analyses, the dependent and independent variables were similarly evaluated to visualise how they correlate with eWBV and which variable could be more strongly correlated with HbA1c.

Ethics considerations: Ethical approval is not required as secondary data was analysed with previous ethics clearance from the Human Research Ethics Committee (HREC) of Charles Sturt University, Australia (re: 2014/158). No primary data were collected from human or animal subject.

## 3. Results

Descriptive statistics show that the routinely performed FBC parameters are essentially within the normal range in the cross-sectional study as shown in Table 1. However, HbA1c is within the abnormal high range indicative of poor glycaemic control. The results further show that lymphocyte ratios are within normal while the platelet ratios are abnormally low except PWR (Table 2).

### 3.1. Question 1: How Does eWBV Correlate with Indices of RBC, Platelet and WBC?

Correlation analysis shows that haemoglobin and haematocrit have unexpectedly different relationships with HbA1c. Mixed correlation is seen among the RBC indices as well as platelet and lymphocytes with both HbA1c and eWBV. Further, RBC count, HCT, and haemoglobin are all positively correlated with eWBV. WBC count shows higher correlation with eWBV compared to platelet count, both positive (Table 3).

### 3.2. Question 2: Which of the Blood Cell Counts and Indices Rank Most Strongly Correlated with Increasing HbA1c?

In the univariate paired *t*-test evaluations of periodic cohort groups, there is an affirmed statistically significant difference in HbA1c between groups (*p* < 0.00001). Among the other 17 variables, only RDW achieves statistical difference (*p* < 0.04)—the level is higher in group 1, i.e., at a period of lower HbA1c or better glycaemic control (Table 4). Correlation analysis to determine the strength of relationships shows that MCV and MCH have relatively stronger but negative correlations with HbA1c (Table 5). Further, MCV, WBC, Platelet count, MLR, and NLR show discrepant relationship by the change scores in Table 4, compared to correlation with the HbA1c in Table 5.

## 4. Discussion

Previous studies in this series on clinical laboratory medicine evaluation of blood flow pathophysiology in diabetes have reported on age and gender factors [47], as well as associations with routine parameters of coagulation profile and dyslipidaemia [48,49]. In this study, focus is on the indices of RBC, platelets ratios, and lymphocyte ratios.

Table 1 is a summary of observation in terms of descriptive statistics. The HbA1c were higher than normal, and this was expected considering that participants were confirmed diabetics. eWBV were within the normal range, which was not expected considering that diabetes is accompanied by raised viscosity due to high glucose concentrations [10,50]. FBC and its indices were normal except for platelets indices of PRR, which may indicate a certain level of risk to bleeding which is not uncommon in diabetes. RPR was also low which may indicate lower risk to adverse outcomes.

By inference, the abnormally high HbA1c reported in this study is indicative of poor glycaemic control. This is consistent to other studies [20,51] and further infers ongoing complex pathophysiological processes that could confound linear relationships with clinical medicine variables.

The normal result for eWBV is not consistent with literature [13], however the dissenting view could be due to variables such as measurement technique, differences in haematocrit, medication, and comorbidity and formula in calculating the eWBV. The indices in this study were normal except for RPR and lymphocyte ratios. Elevated RPR has been reported to worsen outcomes in several diseases, especially acute pancreatitis [52,53], and indicated worse prognosis in spontaneous deep-seated intracerebral haemorrhage [35]. Raised NLR reported to indicate impaired glucose metabolism and higher risk of diabetes, can also be used to monitor diabetes treatment along with HbA1c [37]. Similarly elevated PLR was reported to predict the development of diabetics [54], as well as inflammation, platelet activation, and atherosclerosis [55]. MLR not only predicts diabetes but is also associated with complications and mortality [56]. A low value for RPR reported in this study should therefore indicate a better outcome.

Many studies on lymphocyte ratios appear to focus on intracranial bleeding [57,58]. That is, the potential in clinical medicine for platelet and/or lymphocyte ratios in monitoring individuals undergoing diabetes management has yet to be clearly investigated. Hence, this report focusing on diabetes data is novel especially for clinical medicine research and practice.

The observation on PRR (Table 2) in this study is an indication of normal level, especially highlighting the cross-sectional average being similar to the recommended 1:20 [44]. A study noted that elevated PRR improved mortality in transfusion patient without worsening organ failure [32]. This report supports other studies, except that PWR was reversed.

Therefore it is inferred that the observation of the normal value of PRR in this study could make for better outcome, and this is supported by a report that up to 95% of individuals attending diabetes monitoring show normal eWBV, which could be explained by antiplatelet prophylaxis in diabetes management [50].

Several prior reports show that high PWR not only improves ischaemic stroke with better prognosis [26], but enhances recovery from acute illness such as acute heart failure and myocardial infarction [25]. Another study reported the low WPR to predict poor diagnosis in delayed cerebral ischemia [29]. For RBC indices, RBC and Hb were observed to be higher in good glycaemic control while MCV, MCH, and RDW were higher in poor glycaemic control [20].

The implications for clinical medicine from this study is on insights into the relationship between whole blood viscosity and blood cell indices in diabetes mellitus. These findings possibly have significant implications for clinical practice particularly in the monitoring and management of diabetic patients. Regarding the indices of platelet and lymphocyte ratios, there is a dearth of information on reference ranges, which this report has advanced, e.g., by providing the range for normal values of PRR (Table 2).

Table 3 summarises observations on correlation analysis, and of interest is that haemoglobin and haematocrit show different relationships with HbA1c. Mixed correlations are seen among the RBC indices as well as platelet and lymphocyte ratios with both HbA1c and eWBV. Further, RBC count, HCT, and haemoglobin are all positively correlated with eWBV. WBC count shows higher correlation with eWBV compared to platelet count, although it is noteworthy that both are positive correlations.

The interpretation for clinical medicine lies in the potential usefulness of critically evaluating the entire blood cell counts and indices on routine FBC. In this inference, it is noteworthy that the concept of managing hyperviscosity (i.e., very high eWBV) with erythrocytapheresis, leukapheresis, or thrombocytapheresis is dependent on the associated blood cell count.

With a focus on diabetes, there is evidence of similar variability that has been implied [59]. Further support is the report that a change of up to 5% in HbA1c level is associated with normal MCHC, as observed in this study, and it calls for a comprehensive review of FBC examination [60].

The implications of this finding for clinical laboratory medicine are on health facilities that are limited by resources. Given the plausibility of variations in linear relationships exhibited by haematological indices (especially haematocrit and haemoglobin), it could be erroneous in clinical medicine practice to use haematocrit and haemoglobin interchangeably. Indeed, it has been cautioned that haemoglobin is better than HCT in the clinical laboratory diagnosis of anaemia among renal patients [61]. What this report adds to the discourse is that the correlation of HbA1c with HCT and haemoglobin may show disparity in diabetes.

Table 4 and Table 5 show the level of changes and associated strength of linear relationships among test biomarkers. It is an interesting observation on hypothesis testing that only the change in RDW achieved a statistically significant difference (Table 4; *p* < 0.04). However, it is pertinent to note that the ‘change scores’ for HCT, haemoglobin, and RBC count are positively correlated with those of HbA1c and eWBV. While the ‘change scores’ for the RBC indices also positively correlate with eWBV; MCV and MCH are negatively correlated with HbA1c. Further, RDW is most negligible in the strength of relation and inverse to both HbA1c and eWBV. Whereas RBC is relatively more strongly correlated to HbA1c compared to haematocrit and haemoglobin; the strength of linearity is much stronger but reversed for eWBV (Table 5).

The inference from these observations is a mix of affirmation and conundrum. First, the observation affirms that changes in RBC count among routine FBC parameters are positively associated with increasing HbA1c, while MCV and MCH may be more strongly but inversely associated. Secondly, our hypothesis is rejected based on RDW. Further, the conundrum is RDW that showed statistically significant different change with increasing HbA1c (Table 4) but the strength of linearity is least among all the haematological parameters evaluated (Table 5). Therefore, it is inferred that RBC count has the strongest association with HbA1c, consistent with subclinical blood flow pathophysiology indicated by eWBV.

Based on the literature, PRR is indicated to improve bleeding-related mortality [32]. Therefore, the positive correlations of change in PRR with both HbA1c and eWBV highlights usefulness in diabetes cardiovascular monitoring. Further, there is evidence of no significant differences in haemoglobin and RBC indices between HbA1c groups [60]. However, there is also report of HCT and haemoglobin but not RBC being significantly correlated with HbA1c albeit among apparently pregnant normo-glycaemia [62], while the observations advanced in this report differ partially and based on apparently diabetic individuals. Further, this report is in agreement with the literature indicating RDW being associated with HbA1c [63].

### Implications for Clinical Medicine and Personalised Care

It has been suggested that abnormal HbA1c level requires further investigation of the FBC, especially the RBC indices [60]. What this report contributes is bringing to the fore an update to support the individualised references in medical practice. The relevance of personalised care in clinical medicine for diabetes management has been known [64]. This paper advances the perspective of clinical laboratory medicine around the utilisation of FBC parameters—the variabilities in observations of correlations and statistical significance implies that a case-by-case interpretation of individual patients’ results could be better than a population reference range.

Further, in a recently published review, the authors acknowledge the three physiological processes to include endothelial dysfunction, hypercoagulability and stasis that underpin thrombophilia pathogenesis, i.e., blood flow pathophysiology, and that stasis is the most significant [65]. However, the authors stopped short of indicating WBV as an available test for stasis. Therefore, the additional contribution of this paper is advancing the awareness of eWBV, which is easily extrapolated at no further cost from HCT and serum protein results if routine FBC and liver function tests are performed.

Study limitations: Age and gender have not been evaluated in this study. Previous study has looked at these independent variables [47]. Although, it must be appreciated that gender is explicitly a non-factor in this study because of the paired cohort analysis being periods between same individual. The medication use or duration of diabetes were unavailable in the database. Therefore, comparisons in this study were limited to univariate paired *t*-tests, which may be considered as multiple testing without correction and without adjustment for confounders. Regression analysis was out of scope and would be the subject of another analysis, which was part of a bigger project. Perhaps another limitation is the retrospective design.

## 5. Conclusions

This report extends on our ongoing studies into eWBV in diabetes with a focus on blood cell indices. Currently, it is the RBC indices alone, but neither platelet nor lymphocyte ratios, have been part of routine haematology test report in clinical laboratory medicine. Knowledge of platelet and lymphocyte ratios have been of research interest in areas of intracranial bleeding. Considering the risk of bleeding in the continuum of blood flow pathophysiology in diabetes, this study investigated the relationship between blood cell indices and eWBV in diabetes. The results show promises for the continued use of RBC indices as well as PRR and RPR. The observation around RDW is not clear and will be re-evaluated through regression analysis.

## Figures and Tables

**Table 1:** Descriptive statistics of cross-sectional data.

	Mean	Median	Mode	SD
HbA1c (%)	7.67	7.10	5.70	1.87
eWBV (mPas)	17.18	17.22	17.05	0.90
Hgb (g/L)	148.01	148.00	152.00	15.73
RBC (10^6^/µL)	5.02	5.00	5.30	0.48
HCT	0.45	0.44	0.43	0.04
MCV (fL)	88.91	90.00	91.00	4.14
MCH (pg)	29.55	29.70	29.70	1.79
MCHC (g/dL)	332.55	333.00	331.00	10.97
RDW (%)	13.21	13.00	12.80	1.19
WBC 10^3^/µL	7.85	7.50	7.40	2.16
Platelets 10^3^/µL	264.66	251.00	246.00	66.10
PWR	35.60	34.33	43.06	10.94
PRR *	20.10	19.96	19.51	5.12
RPR	20.11	19.22	21.23	4.98
MLR	0.41	0.26	0.24	1.92
NLR	1.94	1.78	1.58	0.78
PLR	117.66	116.26	69.23	44.23

* It is noteworthy that actual PRR (platelet/RBC, e.g., 150,000:4,700,000) is in the level of 1:0.03. The translation in clinical medicine research and practice is number of RBC per platelet count. Thus, 4,700,000/150,000 would be 14, hence may be expressed as 1:14 [44].

**Table 2:** Representation of observed mean levels with reference ranges.

Variable	Observation (Mean)	Reference Range *
HbA1c%	7.67	<5.7
eWBV mPas	17.18	15.01–19.01
Hgb g/L	148.01	135–175
RBC 10^6^/µL	5.02	4.7–6.1
HCT	0.45	0.37–0.54
MCV (fL)	88.91	80–100
MCH (pg)	29.55	27–33
MCHC g/L	332.55	320–360
RDW%	13.21	11.5–14.5
WBC 10^3^/L	7.85	4.0–11.0
Platelets 10^3^/µL	264.66	150–450
PWR	35.60	20–40
PRR ^†^	1:20.10	1:14–1:31
RPR%	20.11	4.5–11.5
MLR	0.41	0.2–0.4
NLR	1.94	1–3
PLR	117.66	90–180

^†^ Number of RBC per platelet count [44]; * reference range as per generally acceptable in lab.

**Table 3:** Correlation analysis outcome *.

	HbA1c	eWBV
HbA1c	1.000	
eWBV (HSS:208 s^−1^)	−0.019	1.000
Haemoglobin	0.027	0.463
RBC	0.087	0.524
Haematocrit	−0.014	0.491
MCV	−0.192	−0.135
MCH	−0.099	−0.023
MCHC	0.102	0.146
RDW	−0.159	−0.044
WBC	0.049	0.223
Platelets	0.060	0.113
PWR	−0.036	−0.086
PRR	0.007	−0.078
RPR	0.111	0.142
MLR	−0.044	0.139
NLR	0.063	0.093
PLR	0.010	−0.028

* Both dependent and independent variables similarly evaluated to visualise how they correlate with eWBV and HbA1c.

**Table 4:** Comparative descriptive statistics of ‘periodic cohort ^†^’ groups.

Variables	Lower Period	Higher Period	Change Score
HbA1c%	7.020	8.330	1.310 *
eWBV mPas	17.190	17.170	−0.020
Hgb g/L	147.733	148.286	0.552
RBC 10^6^/µL	5.010	5.030	0.020
HCT	0.445	0.446	0.001
MCV (fL)	88.910	88.900	−0.010
MCH (pg)	29.530	29.570	0.040
MCHC g/L	33.200	33.300	0.100
RDW%	13.302	13.111	−0.190 **
WBC 10^3^/L	7.970	7.727	−0.243
Platelets 10^3^/µL	266.133	263.190	−2.943
PWR	35.540	35.660	0.120
PRR ^†^	19.810	20.390	0.580
RPR%	20.090	20.130	0.040
MLR	0.270	0.540	0.270
NLR	1.900	1.970	0.070
PLR	115.440	119.870	4.430

^†^ Period of lower HbA1c level paired with set of when HbA1c, instead of baseline, vs. post-intervention. Paired *t*-test: * *p* < 0.00001, ** *p* < 0.04.

**Table 5:** Correlation coefficients of ‘change scores’ among variables *.

	HbA1c	eWBV
HbA1c	1	
eWBV	−0.07	1
Hgb	0.022	0.657
RBC	0.297	0.539
HCT	0.01	0.639
MCV	−0.471	0.133
MCH	−0.373	0.207
MCHC	0.006	0.198
RDW	−0.001	−0.056
WBC	0.048	0.001
PLT	0.195	−0.083
PWR	0.044	−0.08
PRR	0.025	0.243
RPR	0.185	−0.048
MLR	−0.074	0.12
NLR	−0.031	0.096
PLR	0.016	0.007

* Dependent and independent variables similarly evaluated to visualise the change that quite strongly correlates with eWBV and HbA1c.

## Data Availability

All data generated or analysed during this study are available and included as 3rd dataset of the published repository.
